# Different Forms of AMPA Receptor Mediated LTP and Their Correlation to the Spatial Working Memory Formation

**DOI:** 10.3389/fnmol.2017.00214

**Published:** 2017-07-04

**Authors:** Derya R. Shimshek, Thorsten Bus, Bettina Schupp, Vidar Jensen, Verena Marx, Liliana E. Layer, Georg Köhr, Rolf Sprengel

**Affiliations:** ^1^Department of Molecular Neurobiology, Max Planck Institute for Medical ResearchHeidelberg, Germany; ^2^Research Group of the Max Planck Institute for Medical Research, Institute for Anatomy and Cell Biology, Heidelberg UniversityHeidelberg, Germany; ^3^Letten Centre and GliaLab, Department of Physiology, Institute of Basic Medical Sciences, University of OsloOslo, Norway; ^4^Department of Neurophysiology, Donders Center for Neuroscience, Radboud University NijmegenNijmegen, Netherlands; ^5^Faculty of Medicine, Institute of Anatomy, University of ZurichZurich, Switzerland; ^6^Physiology of Neuronal Networks, Central Institute for Mental Health (CIMH), Medical Faculty, Heidelberg UniversityMannheim, Germany

**Keywords:** AMPA receptors, GluA1, GluA2, *Gria1* knockout mice, *Gria2* knockout mice, long-term potentiation (LTP), spatial working memory (SWM), spatial reference memory (SRM)

## Abstract

Spatial working memory (SWM) and the classical, tetanus-induced long-term potentiation (LTP) at hippocampal CA3/CA1 synapses are dependent on L-α-amino-3-hydroxy-5-methylisoxazole-4-propionate receptors (AMPARs) containing GluA1 subunits as demonstrated by knockout mice lacking GluA1. In GluA1 knockout mice LTP and SWM deficits could be partially recovered by transgenic re-installation of full-length GluA1 in principle forebrain neurons. Here we partially restored hippocampal LTP in GluA1-deficient mice by forebrain-specific depletion of the GluA2 gene, by the activation of a hypomorphic GluA2(Q) allele and by transgenic expression of PDZ-site truncated GFP-GluA1(TG). In none of these three mouse lines, the partial LTP recovery improved the SWM performance of GluA1-deficient mice suggesting a specific function of intact GluA1/2 receptors and the GluA1 intracellular carboxyl-terminus in SWM and its associated behavior.

**Life Science Identifiers:**

urn:lsid:<Sprengel>:< B6N.129-*Gria1^tm1Rsp/J^*                  Mus musculus >:< RRID:IMSR_JAX:019011>

urn:lsid:<Sprengel>:< B6N.129-*Gria2^tm2Rsp^*                     Mus musculus >:< RRID:MGI: 3612255>

urn:lsid:<Sprengel>:< B6N.129-*Gria2^tm3Rsp^*                     Mus musculus >:< RRID:EM: 09212>

um:lsid:<Schutz>:<*Tg^(Camk2a-cre)1Gsc^*                               Mus musculus >:< RRID:MGI:2181422>

urn:lsid:<Mayford>:<*Tg^(Camk2a-tTA)1Mmay/J^*                     Mus musculus >:< RRID:IMSR_JAX:003010>

urn:lsid:<Sprengel>:<*Tg^(tetO–lacZ-GFPGluA1(TG)8.1Rsp)^*     Mus musculus >:< RRID:MGI:submitted>

## Introduction

Changes in synaptic efficacy in the central nervous system are thought to underlie learning and memory. Activity-dependent and input-specific increases in excitatory postsynaptic responses, described as long-term potentiation (LTP) in hippocampal field recordings (Bliss and Lomo, 1973), have served as an attractive cellular correlate of hippocampus-dependent behavior. At hippocampal CA3-to-CA1 synapses, LTP induction requires the N-methyl-D-aspartate receptors (NMDARs; Collingridge et al., 1983; Coan et al., 1987; Errington et al., 1987; Tsien et al., 1996; Bannerman et al., 2012). The NMDAR activation is followed by a long lasting increase of L-α-amino-3-hydroxy-5-methylisoxazole-4-propionate receptor (AMPAR) currents. The currents are mediated by abundant heteromeric GluA1/2 and by minor populations of GluA2/3 AMPARs (Petralia and Wenthold, 1992; Wenthold et al., 1996). The AMPAR subunit GluA4 is expressed only transiently in CA1 pyramidal neurons, namely while synaptic connectivity is forming, and is not involved in AMPAR-mediated signal transmission in hippocampal pyramidal neurons of adult mice (Monyer et al., 1991; Zhu et al., 2000; Luchkina et al., 2017). In absence of genetically removed GluA1 – 3 subunits no AMPAR currents could be measured in CA1 pyramidal cells of adult mice (Lu et al., 2009).

Gene-targeted mice deficient for the AMPAR subunit GluA1 (GluA1 knockout; *Gria1*^−/−^) have revealed an essential role for GluA1 in hippocampal LTP at Schaffer collateral/CA1 synapses. Thus, the CA3-to-CA1 LTP was strongly impaired in absence of GluA1 (Zamanillo et al., 1999; Hoffman et al., 2002; Jensen et al., 2003), and transgenic expression of GFP-tagged GluA1 in CA1 pyramidal neurons of *Gria1*^−/−^ mice partially restored the CA3-to-CA1 LTP (Mack et al., 2001). Moreover, the dramatic loss of GluA2 dendritic immunosignal in hippocampi of *Gria1*^−/−^ mice suggested several important functions for AMPAR subunits at mature CA1 synapses. Firstly, GluA1 is necessary for the huge pool of extra-synaptic AMPARs. Secondly, the extra-synaptic AMPARs are composed of GluA1/2 receptors. Thirdly, the minor pool of the GluA2/3 receptors is sufficient for regular synaptic transmission, suggesting that the extra-synaptic AMPAR pool is recruited for increased, LTP-mediated synaptic transmission. Finally, GluA2 homomeric receptors are poorly translocated to dendritic membranes (Zamanillo et al., 1999).

Subsequent intensive research, analyzing the subunit composition of AMPARs in detail, led to a widely accepted model for the role of AMPAR subtypes in synaptic transmission and synaptic plasticity (for reviews see Derkach et al., 2007; Henley and Wilkinson, 2016). According to this model the Q/R site editing of the GluA2 subunit is essential for the formation of Ca^2+^-impermeable AMPAR assemblies (Sommer et al., 1991). The GluA2/3 AMPARs maintain basal synaptic transmission. In contrast, extra-synaptic GluA1/2-containing AMPARs are actively translocated into potentiated synapses upon LTP induction (Hayashi et al., 2000; Shi et al., 2001). Immediately after LTP induction, Ca^2+^-permeable AMPARs are incorporated into the synapses (Plant et al., 2006; Rozov et al., 2012), (but see Adesnik and Nicoll, 2007) which might facilitate LTP expression. Due to their somatic and intracellular accumulation, GluA2 homomeric receptors contribute only poorly to AMPAR mediated signaling (Greger et al., 2002).

Unexpectedly, *Gria1*^−/−^ mice showed a normal spatial reference memory (SRM) in the Morris Water Maze despite the absence of field-LTP (Zamanillo et al., 1999; Reisel et al., 2002). Other genetic mouse models failed likewise to reveal a strong correlation between hippocampal LTP and hippocampus-dependent learning (Shimshek et al., 2006; Neves et al., 2008; Wiltgen et al., 2010; Bannerman et al., 2012). These findings raised doubts concerning the importance of hippocampal LTP in SRM as discussed by several authors (Bliss and Lomo, 1973; Morris et al., 1986; Tsien et al., 1996; Malenka and Nicoll, 1999; Malenka and Bear, 2004).

Despite the normal SRM of *Gria1*^−/−^ mice a robust impairment in the rewarded alternation task on the elevated T-maze—the standard behavioral test for the spatial working memory (SWM) performance in rodents (Rawlins and Olton, 1982; Deacon et al., 2002)—was detected in *Gria1*^−/−^ mice (Reisel et al., 2002). This SWM deficit was directly correlated to the LTP impairment, as shown by the partial restoration of SWM and LTP in *Gria1*^−/−^ mice that express GFP-tagged-GluA1 in principal forebrain neurons (Mack et al., 2001; Schmitt et al., 2005).

A recent study showed that AMPAR-mediated CA3-to-CA1 LTP is not strictly GluA1 dependent but requires a reserve pool of extra-synaptic ionotropic glutamate receptors (iGluRs; Granger et al., 2013). An increased surface expression of Ca^2+^-permeable iGluRs provided e.g., by the Q/R site unedited, trafficking competent GluA2(Q), a kainate receptor GluK1 or C-terminally truncated GluA1, was sufficient to restore LTP at mature CA1 synapses in absence of the endogenous AMPAR subunits (GluA1–3; Granger et al., 2013). Similarly, PDZ-site truncated GluA1 was sufficient for CA1 LTP as reported for gene targeted mice (Kim et al., 2005).

We noticed in previous studies that CA3-to-CA1 LTP is not necessarily linked to the SWM performance. The forebrain-specific depletion of GluA2 in *Gria2*^Δ*Fb*^ mice was associated with SWM impairment although CA3-to-CA1 LTP was well-developed (Shimshek et al., 2006). Similarly, the transgenic expression of PDZ-site truncated GFP-GluA1(TG) was comparable to the GFP-GluA1 expression, but the GFP-GluA1(TG) expression could not rescue the SWM impairment in GluA1 deficient mice (Freudenberg et al., 2013a,b). To further dissect AMPAR functions in LTP and SWM, we genetically activated AMPARs containing homomeric GluA3, heteromeric GluA2(Q)/3 or PDZ-site truncated GFP-GluA1(TG) in principal forebrain neurons of *Gria1*^−/−^ mice and analyzed AMPAR subunit expression, pairing-induced and field-LTP and the SWM of the three different mouse lines.

## Materials and methods

### Ethical statement

Experiments were performed according to the institutional guidelines of the Max Planck Society and of the animal core facility (IBF) of the Heidelberg University. These guidelines adhere to the German Animal Welfare Act: Regulation for the Protection of Animals Used for Experimental or Other Scientific Purposes (Animal Welfare Regulation Governing Experimental Animals (TierSchVersV). Animal numbers for molecular and histological experiments were recorded under the protocol MPI/T-6/06; 15/08; 20/; 28/11. Genetic manipulations and behavioral experiments were licensed by the Regional Council in Karlsruhe, Germany (35-9185.81/G-4/02 and 35-9185.81/G-71/10). Efforts were made to minimize the number of animals used.

### Mouse lines

For the generation of *Gria1*^−/−^*/2*^Δ*Fb*^, *Gria1*^−/−^*/2*^*QFb*^, and *Gria1*^−/−^*/Tg8.1* mice the following gene-targeted and transgenic mouse lines were used as founder lines:

Gene-targeted mice: ***Gria1***^−/−^ (*Gria1*^*tm1Rsp*^, Zamanillo et al., 1999 IMSR_JAX:019011); ***Gria2***^+/***neo***^ (*Gria2*^*tm2Rsp*^, Feldmeyer et al., 1999 MGI: 2178121); ***Gria2***^***2lox***^ (*Gria2*^*tm3Rsp*^; Shimshek et al., 2005 MGI:3612398).
Transgenic mice: ***Tg***^***Cre4***^ (*Tg*^*(Camk2a-cre)1Gsc*^; Mantamadiotis et al., 2002 MGI:4839474); ***Tg***^***8.1***^ (*Tg*^(*tetO-lacZ-GFPGluA*1(*TG*)*8.1Rsp*^; Freudenberg et al., 2013a MGI:submitted + *Tg*^*aCaMKII-tTA*^ (*Tg*^(*Camk*2*a-tTA*)1*Mmay*^; Mayford et al., 1996 MGI:4844270)).

### Breeding schemes

***Gria1^−/−^/2^ΔFb^*** *(Gria1*^−/−^/*Gria2*^*2lox*^*/Tg*^*Cre4*^*)*: *Gria1*^+/−^*/Gria2*^+/*lox*^X *Gria1*^+/−^/*Gria2*^+/*lox*^/*Tg*^*Cre4*^. Littermates with the genotypes *Gria1*^+/+^*/2*^+/+^, *Gria1*^+/+^*/2*^+/*lox*^ or *Gria1*^+/+^*/2*^*2lox*^ were used as wild-type controls, *Gria1*^−/−^*, Gria1*^−/−^*/Tg*^*Cre4*^, *Gria1*^−/−^*/2*^+/*lox*^ or *Gria1*^−/−^*/2*^*lox*/*lox*^ were used as GluA1 deficient mice in behavioral experiments.
***Gria1^−/−^/2^QFb^*** *(Gria1*^−/−^*/Gria2*^+/*neo*^*/Tg*^*Cre4*^*)*: *Gria1*^+/−^*/Gria2*^+/*neo*^ X *Gria1*^+/−^*/Tg*^*Cre4*^. Littermates with the genotypes *Gria1/2*^+/+^ or *Gria1*^+/+^*/Tg*^*Cre4*^ were used as wild-type controls, *Gria1*^−/−^*/Tg*^*Cre4*^ or *Gria1*^−/−^*/2*^+/+^ were used as GluA1 deficient mice in behavioral experiments.
***Gria1^−/−^/Tg8.1*** (*Tg*^*aCaMKII-tTA*^/*Tg*^(*tetOhbox*−*lacZ-GFPGluA*1(*TG*)8.1*Rsp*):^ *Gria1*^+/−^*/Tg*^*aCaMKII-tTA*^ X *Gria1*^+/−^/*Tg*^(*tetO-lacZ-GFPGluA*1(*TG*)8.1^. As controls *Gria1*^+/+^ were used.

### Genotyping

Mice were genotyped by tail-PCR with specific primers. Indicated below are the names of primers, primer sequences, and the approximate lengths of the amplified gene fragments.

***Gria1***^−/−^:1005 (5′-AAT GCC TAG TAC TAT AGT GCA CG-3′), 3′intro3 (5′-CTG CCT GGG TAA AGT GAC TTG G-3′), 2X1Lox-pz (5′-CAC TCA CAG CAA TGA AGC AG-3′), *Gria1*^+^: 191 bp and *Gria1*^−^: 265 bp.
***Gria2***^+/***neo***^: MH60 (5′-CAC TCA CAG CAA TGA AGC AGG AC-3′), MH53a (5′-GAA TGT TGA TCA TGT GTT TCC CTG-3′) and MH117 (5′-GTT CGA ATT CGC CAA TGA CAA GAC G-3′), *Gria2*^+^: 500 bp and *Gria2*^*neo*^: 400 bp.
***Gria2***^***2lox***^: VM12 (5′-GCG TAA GCC TGT GAA ATA CCT G-3′) and VM10 (5′-GTT GTC TAA CAA GTT GTT GAC C-3′), *Gria2*^+^: 250 bp and *Gria2*^*lox*^: 350 bp.
***Tg***^***(tetO-lacZ, -GFPGluA1(TG)8.1)***^: VM-70 (TGG GAG CCA CAG GAT AAA AGC) and VM-72 (GTG AGC CAA GAT TGT GCC ACT GC) to amplify a 286 bp DNA fragment.
***Tg***^***Cre4***^*:* rspCre1 (5′-ACC AGG TTC GTT CAC TCA TGG-3′) and rspCre2 (5′-AGG CTA AGT GCC TTC TCT ACA C-3′) to amplify a 200 bp DNA fragment.
***Tg***^***aCaMKII-tTA***^: Ca25: GCT CAG AAG CCC CAA GCT CG and CAs25as: CAG CGC CTA ACT CTG GAC AC and Casli 3: TAA GCA GCT CTA TGC GCT GTT A to amplify a PCR fragment in wild-type of 380 bp and transgenic mice of 500 bp (Freudenberg et al., 2013a).

### Immunohistochemistry

Coronal 70–100 μm thick vibratome sections were analyzed using different primary antibodies as described (Shimshek et al., 2005, 2006). Anti-Cre recombinase (1:3,000, polyclonal, gift from G. Schuetz; licensed from Covance, RRID:AB_11220031), anti-GluA1 (1:600, polyclonal, RRID:AB_390157) and anti-GluA2 (1:50, polyclonal, Millipore, RRID:AB_2336198) in combination with secondary anti-mouse (RRID:AB_2336176) and anti-rabbit (RRID:AB_2313567) antibodies coupled to horseradish-peroxidase (Vector Laboratories, each 1:600) or with biotinylated secondary antibodies (Vector Laboratories, 1:600, RRID:AB_2313581; RRID:AB_2313606) and ABC-peroxidase kit (Vector Laboratories, RRID:AB_2336827) were used.

### Immunoblots

Mouse brains were removed and both hippocampi were isolated. Total protein was prepared and immunoblots were performed as described (Mack et al., 2001). Antibodies used: anti-GluA1 (1:2,000, polyclonal, Millipore, RRID:AB_390157), anti-GluA2 (1:800, monoclonal, Millipore, clone L21/32, RRID:AB_10806492), anti-GluA3 (1:1,000, monoclonal, Millipore, clone 3B#, RRID:AB_2113897), anti-GluA4 (1:400, polyclonal, Millipore, RRID:AB_310095), anti-GluN1 (1:600, polyclonal, Millipore, RRID:AB_2112158), anti-β actin (1:40,000, monoclonal, Sigma, clone AC-15, RRID:AB_476744); secondary goat anti-rabbit (RRID:AB_2336198) and goat anti-mouse coupled to horseradish-peroxidase (1:15,000, Vector; RRID:AB_2336171). Data are presented as mean ± SEM. Western blot quantification was statistically evaluated by analysis of variance (ANOVA) measurements followed by Holm-Sidak's multiple comparison and Bonferroni *post-hoc* tests (Prism 6, RRID:SCR_002798; IGOR Pro, RRID:SCR_000325).

### Current-voltage-relations

Brains were removed from deeply anesthetized mice (halothane; age P42) and transverse hippocampal slices (250 μm) were prepared and incubated for 30 min at 37°C in artificial CSF (ACSF) containing (in mM): 125 NaCl, 25 NaHCO_3_, 2.5 KCl, 1.25 NaH_2_PO_4_, 1 MgCl_2_, 25 D-glucose, 2 CaCl_2_; bubbled with 95% O_2_/5% CO_2_ (pH 7.4). Patch pipettes were pulled from borosilicate glass capillaries and had resistances of 4–7 MΩ when filled with (in mM) 125 Cs-gluconate, 20 CsCl, 10 NaCl, 10 HEPES, 0.2 EGTA, 4 MgATP, 0.3 Na_3_GTP, 100 μM spermine, and 2.5 mM QX-314 (pH 7.3, 290–305 mOsm). All chemicals were obtained from Sigma. Series resistances and input resistances were continuously monitored by measuring peak and steady-state currents in response to hyperpolarizing pulses (−5 mV; 20 ms). Liquid junction potentials were corrected. Synaptic currents were activated between −70 and +40 mV in 10 mV steps by stimulating the Schaffer collateral/commissural fibers in *str. radiatum* 150 μm away from the CA1 cell body with a glass electrode filled with 1 M NaCl. AMPAR currents were recorded in presence of 50 μM D-2-amino-5-phosphonopentanoic acid (D-AP5; Tocris), 10 μM bicuculline methiodide (Sigma) and 1 μM CGP 55845 (Tocris). Single traces were analyzed and illustrated. The rectification index (RI) is given as the current ratio at +40 and −60 mV. Data are presented as mean ± SEM. Statistical significance was evaluated by a two-tailed, unpaired Student's *t*-test.

### Low frequency induced LTP in whole-cell recordings

Pairing-induced LTP was induced by pairing low frequency stimulation (120 pulses, 0.67 Hz) with postsynaptic depolarization to 0 mV for 3 min as published in Chen et al. (1999). Monopolar stimulation electrodes were placed in the *str. radiatum* and in the *str. oriens*. The former was used to induce LTP, whereas the latter activated the control pathway. Excitatory postsynaptic currents (EPSCs) were elicited by activation of the two pathways (0.2 Hz) and were recorded for 20 min at –70 mV after the LTP-induction. The following intra- and extra-cellular solutions were used: Intracellular (in mM): 120 CsGluconate, 10 CsCl, 8 NaCl, 10 HEPES, 10 phosphocreatine, 0.2 EGTA, 4 MgATP, 0.3 NaGTP. The pH was set to 7.24 with CsOH and osmolarity was analyzed (295–310 mOsm). Extracellular (in mM): 124 NaCl, 26 NaHCO_3_, 2.5 KCl, 1.25 NaH_2_PO_4_, 4 MgSO_4_, 4 CaCl_2_, 10 glucose. All chemicals were obtained from Sigma. Statistical analysis was done by a two-tailed paired Student's *t*-test.

### Tetanus induced LTP in hippocampal field recordings

Potentiation of hippocampal field excitatory postsynaptic potentials (EPSPs) was induced by tetanic stimulation as previously published. In all these studies Vidar Jensen and Øivind Hvalby performed the experiments under the same conditions and at the same E-Phys. setups (Feldmeyer et al., 1999; Zamanillo et al., 1999; Mack et al., 2001; Jensen et al., 2003; Shimshek et al., 2006). To standardize tetanization strength in different experiments, the tetanic stimulation strength was set in response to a single shock at intensity just above the threshold for generating a population spike. Synaptic efficacy was assessed measuring the slope of the fEPSP in the middle third of its rising phase. Six consecutive responses (1 min) were averaged and normalized to the mean value recorded 4–7 min prior to tetanic stimulation. In some experiments D-AP5 (50 μM, Sigma) was present during the recordings. Statistical significance of LTP levels between tetanized and non-tetanized pathways were calculated by Student's paired two-tailed *t*-test. LTP levels between genotypes were evaluated by linear mixed model statistical analysis (SAS 9.2, RRID:SCR_008567).

### Spatial working memory in rewarded alternation on a T-maze (non-matching-to-place paradigm)

SWM was administrated in the rewarded alternation task on an elevated T-maze (Deacon et al., 2002; Reisel et al., 2002). The T-maze consisted of a start arm (47 × 10 cm) and two identical goal arms (35 × 10 cm) with 10 cm high walls made out of black-painted wood. Mice were kept on diet at 85–90% of the starting body weight and were habituated to the investigator and the T-maze 2 days before testing. For the test, each trial consisted of a sample run followed by a choice run; the two separated by 15 s. During each run a food reward (30 μl sweetened, condensed milk; 4% fat, 10% fat-free dry milk, 27% sugar) was available in a food vial at the end of both arms. On the sample run the choice arms was blocked and the mouse picked up the reward in the sample arm. For the choice run, both arms of the T-maze were open and mice were rewarded for choosing the choice arm and unrewarded when choosing the previously visited sample arm. Correct choices in the choice runs of eight trials per day (four trials in the morning and four trials in the afternoon) were pooled and monitored as daily “block” performance. Behavior was statistically evaluated by analysis of variance (ANOVA) measurements followed by Holm-Sidak's multiple comparison and Bonferroni *post-hoc* tests (Prism 6, RRID:SCR_002798; IGOR Pro, RRID:SCR_000325).

### Spatial reference memory on an elevated Y-maze (non-matching-to-place paradigm)

Acquisition of SRM was performed with mice kept on a strict food diet (remain to 85–90% of the starting body weight) on an elevated Y-shaped maze with prominent extra-maze cues as previously described (Shimshek et al., 2006). In brief, the Y-maze consisted of three identical arms without walls (arms: 50 × 10 × 0.5 cm; angle: 120°; height: 110 cm) made of black painted wood. Mice were trained in 10 sessions per day (inter-trial interval of 10–15 min; 10 sessions in total) to find a milk reward (30 μl sweetened milk) at the end of a designated target arm (marked by a checkerboard pattern as extra-maze cue). The other two arms were assigned as starting position in a pseudo-random order (no more than three successive starts from the same arm with equal numbers of starting positions per day). On a given trial, the mouse was placed at the distal end of the starting arm and the initial entering of one of the other two arms was evaluated as correct (target arm) or incorrect (other start arm) trial. During the initial two sessions, exploring the maze and consuming the bait in the target arm (including entering and re-entering of all arms) was allowed to habituate to the spatial reward location. From session three on, the mouse was removed from the Y-maze when entering the wrong arm. To avoid any olfactory, visible or tactile cue inside the setup directed to a particular arm, the Y-maze was rotated by 120° in random direction between each trial. Mice were trained in two daily blocks of five trials (one in the morning, the other in the afternoon) for 10 days (100 trials in total). Successful trials were recorded and pooled as daily performance. Data represent mean ± SEM. Behavior was statistically evaluated analysis of variance (ANOVA) measurements followed by Holm-Sidak's multiple comparison and Bonferroni *post-hoc* tests (Prism 6, RRID:SCR_002798; IGOR Pro, RRID:SCR_000325).

## Results

### Expression of Ca^2+^-permeable AMPARs in GluA1-deficient mice

Restoration of endogenous Ca^2+^-permeable AMPARs in GluA1-deficient mice (*Gria1*^−/−^) was achieved in *Gria1*^−/−^*/2*^Δ*Fb*^ and *Gria1*^−/−^*/2*^*QFb*^ mice either by Cre-mediated deletion of the *Gria2*^*2lox*^ gene (Shimshek et al., 2006) or Cre-mediated removal of a loxP-flanked selection marker in *Gria2*^*neo*^. The presence of the neo gene in the targeted *Gria2* gene attenuates the expression of the GluA2 Q/R-site editing-deficient, hypomorphic *Gria2*^*neo*^ allele (Feldmeyer et al., 1999). The αCaMKII promoter-driven transgene *Tg*^*Cre4*^ (Mantamadiotis et al., 2002) was used to provide specific Cre expression in principal neurons of the forebrain.

The *Gria1*^−/−^*/2*^Δ*Fb*^ mice are viable in contrast to *Gria1*/2 double knockout mice, which die shortly after birth (V. Mack, personal observation). *Gria1*^−/−^*/2*^*QFb*^ mice were also viable, but the epileptic phenotype observed in mice with forebrain-specific, heterozygous GluA2(Q) expression (*Gria2*^*QFb*^; Krestel et al., 2004), persisted in the absence of GluA1. However, life expectancy of *Gria1*^−/−^*/2*^*QFb*^ mice was increased, thus permitting behavioral analysis; more than 60% of *Gria1*^−/−^*/2*^*QFb*^ mice reached P60 (Supplementary Figure 1) compared to <40% of *Gria2*^*QFb*^ mice (Krestel et al., 2004).

**Figure 1:**
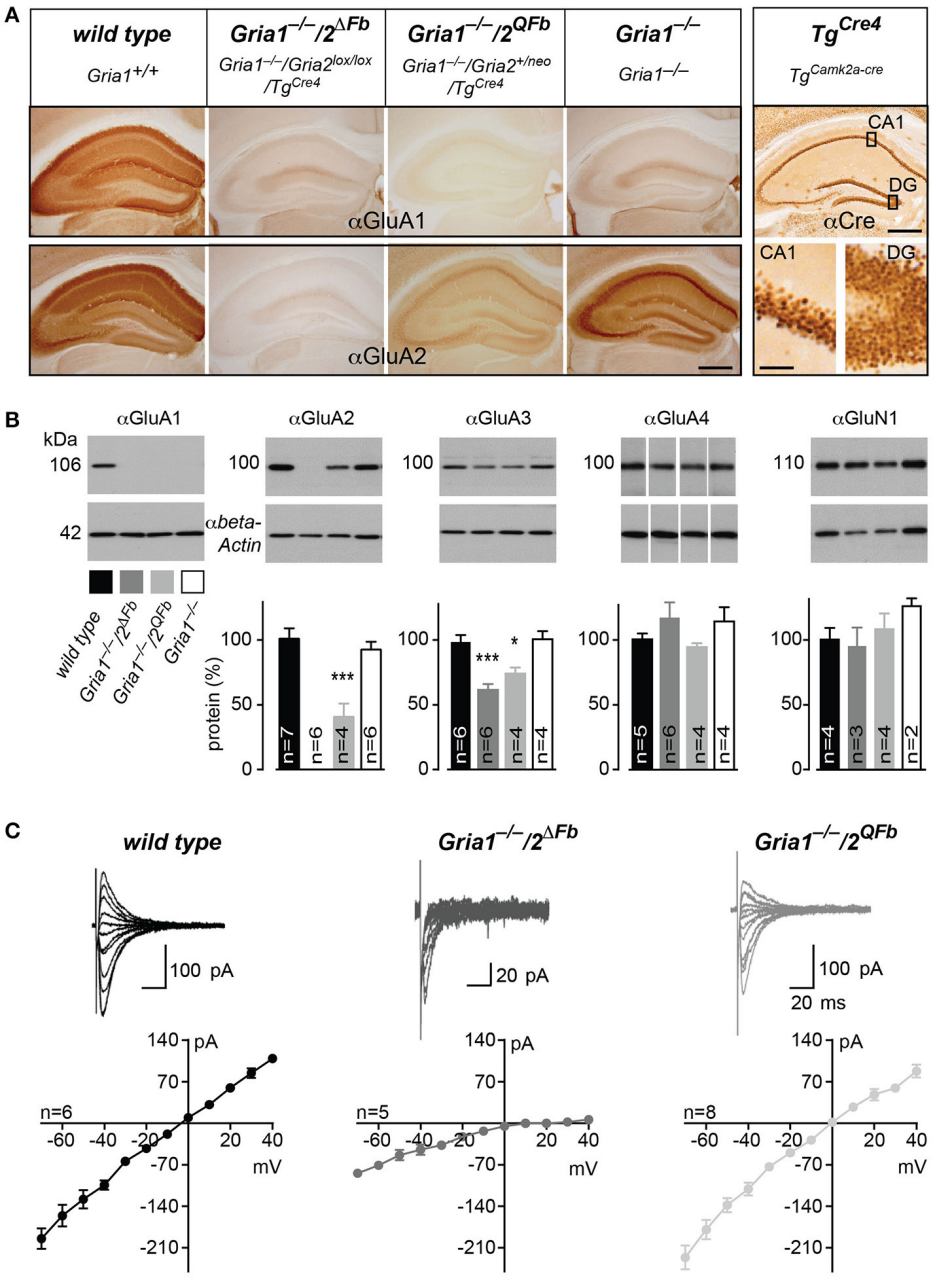
Activation of Ca^2+^-permeable AMPARs in *Gria1*^−/−^ mice by additional manipulations of the *Gria2* gene. **(A)** Immunohistochemically stained hippocampi for GluA1 (top panel) and for GluA2 (bottom panel) from wild-type, *Gria1*^−/−^*/2*^Δ*Fb*^, *Gria1*^−/−^*/2*^*QFb*^, and *Gria1*^−/−^ mice. Anti-Cre immunostaining (right box) of the hippocampus (top) and in higher magnification of the principal cell layers *Cornu Ammonis* area 1 (CA1) and dentate gyrus (DG, bottom) from *Tg*^*Cre4*^ mice (*Tg*^*aCaMKII-Cre*^) employed for forebrain-specific *Gria2* gene manipulation. Scale bars: hippocampus, 500 μm; sublayers, 50 μm. (**B**) Immunoblotting against GluA1, 2, 3, 4, and GluN1 (top panel) from hippocampal whole protein lysates of adult wild-type (black), *Gria1*^−/−^*/2*^Δ*Fb*^ (dark gray), *Gria1*^−/−^*/2*^*QFb*^ (light gray) and *Gria1*^−/−^ mice (white). Anti-β-actin immunosignals (bottom panel) were used for normalization of protein levels relative to wild type (normalized protein in %, diagram). Data in mean ± SEM. ^*^p < 0.05, ^***^p < 0.005. **(C)** I/V relationships and representative AMPAR-mediated currents at different holding potentials of wild-type (black), *Gria1*^−/−^*/2*^Δ*Fb*^ (dark gray) and *Gria1*^−/−^*/2*^*QFb*^ mice (light gray). Numbers of used animals are depicted/added in the diagrams.

To quantify and to visualize the expression of AMPAR subunits, we determined the hippocampal expression pattern of GluA1–3. As expected from the Cre expression pattern of *Tg*^*Cre4*^ mice, the hippocampal GluA2 expression was abolished in *Gria1*^−/−^*/2*^Δ*Fb*^. In *Gria1*^−/−^*/2*^*QFb*^ mice the GluA2 signal was reduced and accumulated at somatic sites as it does in *Gria1*^−/−^ mice (Figure 1A). The normalized protein levels in immunoblots of hippocampal extracts confirmed the absence of GluA2 in *Gria1*^−/−^*/2*^Δ*Fb*^. The strong GluA2 reduction in *Gria1*^−/−^*/2*^*QFb*^ compared to *Gria1*^−/−^ mice (40.8 ± 10.4% vs. 92.8 ± 6.1%, mean ± SEM, *p* < 0.005; Figure 1B) was more pronounced than described for the expression of the modified *Gria2*^*neo*^ gene (Feldmeyer et al., 1999). The reduction and lack of GluA2 was accompanied by a substantial reduction of GluA3 in *Gria1*^−/−^*/2*^Δ*Fb*^ (74.3 ± 4.6%) and *Gria1*^−/−^*/2*^*QFb*^ mice (61.7 ± 4.5%) compared to control (98.7 ± 5.4%) and *Gria1*^−/−^ mice [100.8 ± 6.4%; *F*_(3, 16)_ = 13.88; *p* = 0.0001; Holm-Sidak pairwise comparison at *p* < 0.03 for *Gria1*^−/−^*/2*^*QFb*^, resp. *p* < 0.0006 for *Gria1*^−/−^*/2*^Δ*Fb*^ vs. *Gria1*^−/−^ and control]. In *Gria1*^−/−^*/2*^*QFb*^ mice GluA3 levels seemed to be more reduced. However, the difference between GluA3 levels in *Gria1*^−/−^*/2*^Δ*Fb*^ and *Gria1*^−/−^*/2*^*QFb*^ did not reach statistical significance (*p* = 0.22). The levels of the NMDAR subunit GluN1 [*F*_(3, 9)_ = 0.952; *p* = 0.456] were not altered. Similarly, the amount of the GluA4 subunit, which is not expressed in mature hippocampal pyramidal neurons, was unchanged [*F*_(3, 15)_ = 1.177; *p* = 0.352].

In order to show that the remaining GluA3 and the activated GluA2(Q) subunits form Ca^2+^-permeable AMPARs in hippocampal pyramidal cells, we performed whole-cell recordings in acute hippocampal slices of *Gria1*^−/−^*/2*^Δ*Fb*^ and *Gria1*^−/−^*/2*^*QFb*^ mice. In accordance with previous studies of homomeric GluA3 AMPARs (Boulter et al., 1990), we observed an increased AMPAR-mediated conductance in hippocampal brain slices of *Gria1*^−/−^/*2*^Δ*Fb*^ (8.02 ± 0.84 pA/V, *n* = 10) compared to *Gria1*^−/−^ (5.13 ± 0.9 pA/V, *n* = 7; *p* < 0.05). In *Gria1*^−/−^*/2*^*QFb*^, the presence of the higher conducting GluA2(Q)-containing AMPARs (Feldmeyer et al., 1999) generated excitatory postsynaptic currents (EPSCs, which were similar to those of AMPARs in slices of wild-type mice (wild type: 12.72 ± 1.96 pA/V, *n* = 15 vs. *Gria1*^−/−^*/2*^*QFb*^ 11.53 ± 3.45 pA/V, *n* = 3; *p* = 0.58). Importantly, both genotypes expressed Ca^2+^-permeable AMPARs in CA1 pyramidal cells, as indicated by rectification indices (RIs) of current-voltage relationships (Figure 1C; Burnashev et al., 1992). In wild-type mice the RI was close to 1 (1.45 ± 0.17), since GluA2 renders AMPARs impermeable for Ca^2+^ (Burnashev et al., 1992). The GluA2-deficiency in *Gria1*^−/−^*/2*^Δ*Fb*^ was confirmed by the RI increase (15.7 ± 6.8 vs. 1.45 ± 0.17, *p* < 0.05), which is characteristic of Ca^2+^-permeable AMPARs lacking the GluA2 subunit (Washburn and Dingledine, 1996). The smaller, but still significant RI increase in *Gria1*^−/−^*/2*^*QFb*^ (2.12 ± 0.15, *p* < 0.05) can be explained by the presence of two G*ria2* alleles (*Gria2* and *Gria2*^Δ*ECS*^) leading to a mixed AMPAR population containing Ca^2+^-permeable GluA2(Q) and Ca^2+^-impermeable GluA2 receptors.

The mixed AMPAR population could also be monitored in brain slices from *Gria1*^−/−^*/2*^*QFb*^ mice by a small but significant amount of NMDAR-independent LTP (1.12 ± 0.03 vs. 1.00 ± 0.02, *p* < 0.01) measured in the presence of the NMDAR antagonist D-AP5 (Supplementary Figure 2) as previously reported in for heterozygous *Gria2*^+/Δ*ECS*^ mice (Feldmeyer et al., 1999). In *Gria1*^−/−^*/2*^Δ*Fb*^ mice the remaining AMPARs resulted in five-fold reduced currents (Figure 1C) and LTP was completely blocked in the presence of the NMDAR antagonist D-AP5 (1.04 ± 0.03 vs. 1.01 ± 0.04, *p* = 0.53; Supplementary Figure 2) as described before for forebrain-specific GluA2 knockout mice (*Gria2*^Δ*Fb*^) mice (Shimshek et al., 2006).

### Ca^2+^-permeable AMPARs and C-terminally truncated GluA1 restore LTP in GluA1-deficient mice partially

**Figure 2:**
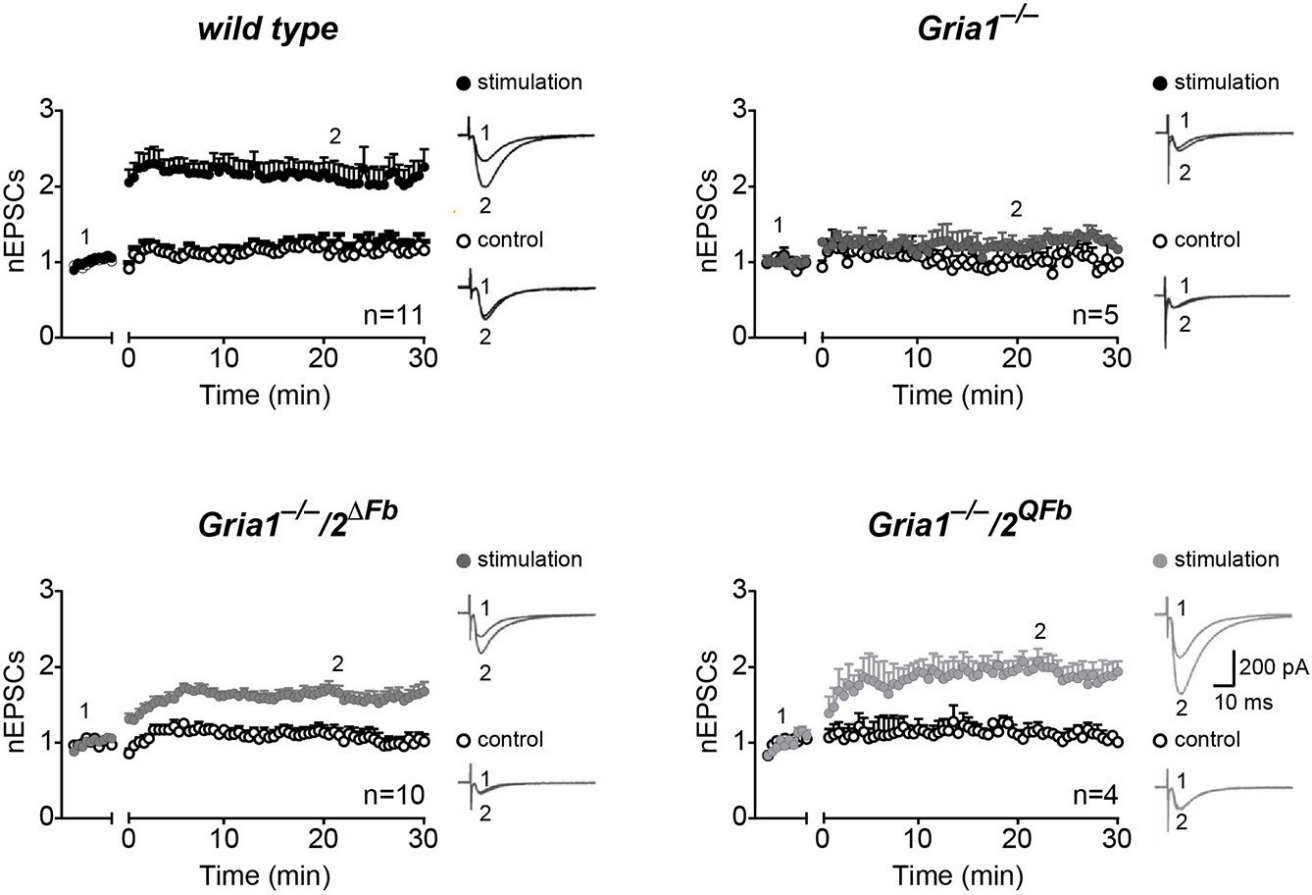
Restoration of hippocampal, pairing-induced LTP. Normalized excitatory postsynaptic potentials (nEPSCs) before (1) and 30 min after (2) applying low frequency pairing (time gap) to stimulation pathways (filled circles) but not to control pathways (open circles). Traces show cellular responses in paired (stimulation, top) and un-paired pathways (control, bottom) from single experiments. Genotypes and numbers of experiments (*n*) are indicated. Scale bars: 10 ms, 200 pA. Data in mean ± SEM. Data from control animals are labeled as wild type.

**Figure 3:**
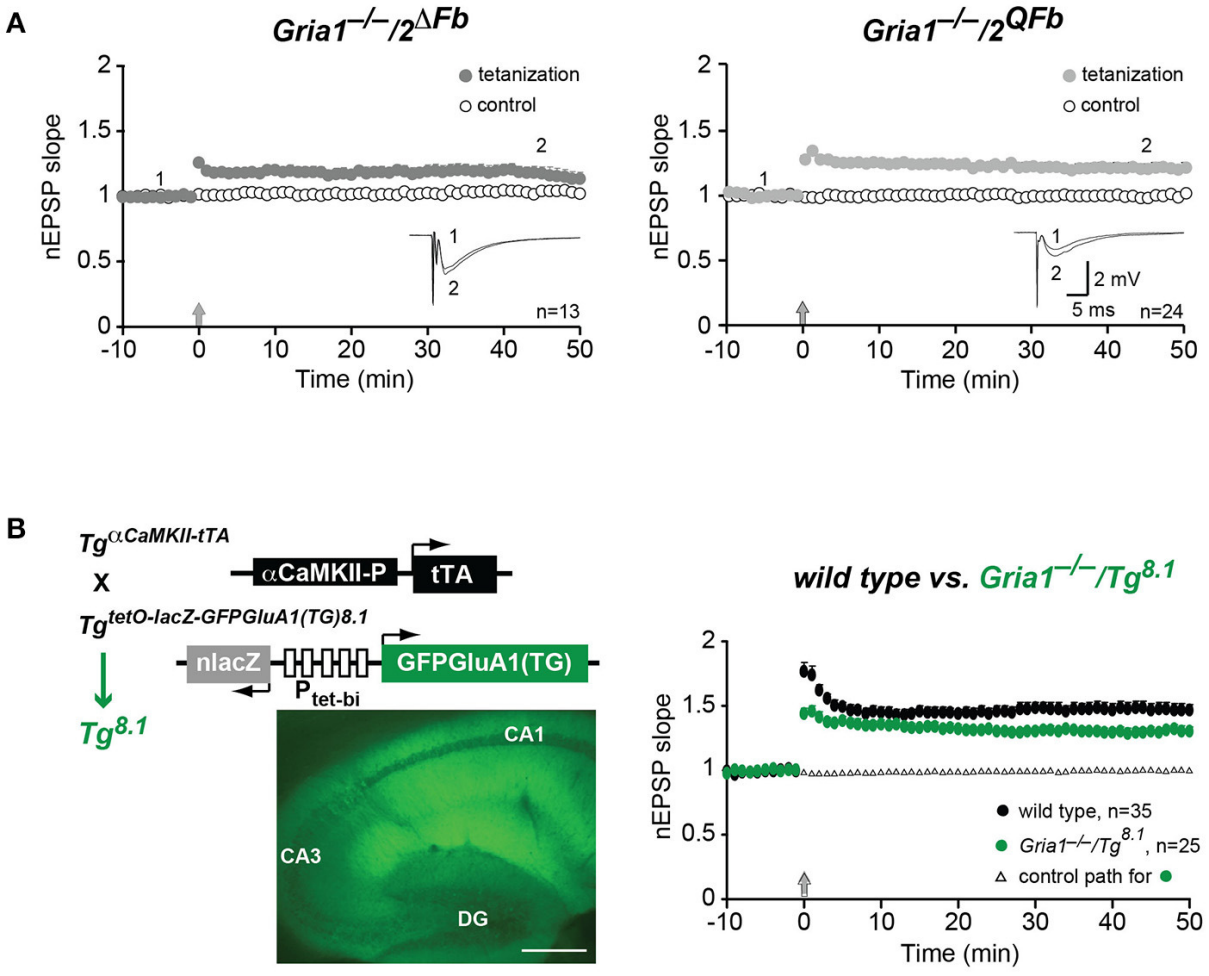
Restoration of hippocampal field-LTP. **(A)** Normalized slopes of excitatory postsynaptic potentials (nEPSP slopes) before (1) and 45 min after (2) applying tetanization (arrows) to stimulation pathways (filled circles) in hippocampal field recordings. Responses from un-tetanized pathway (open circles) serve as control. Numbers of experiments (*n*) are indicated. Insets: mean of six consecutive synaptic responses from single experiments. Scale bars: 5 ms, 2 mV. **(B)** LTP restoration at CA3-to-CA1 synapses by transgenic expression of C-terminally truncated GFP-GluA1(TG) in *Gria1*^−/−^ mice (*Gria1*^−/−^*/Tg8.1*). In GFP-GluA1(TG) the C-terminal Leucine deletion of GluA1 destroys the GluA1 carboxy-terminal PDZII-like motif (TGL) (Freudenberg et al., 2013a). (**B**, left) Cartoons of transgenes encoded by *Tg8.1* mice. Transgene *Tg*^*aCaMKII-tTA*^ restricts the transgenic tTA expression to principal cells of the forebrain by a promoter fragment of the αCaMKII gene. The transgene *Tg*^*nlacZtetOGFPGluA*1(*TG*)^ enables tTA-dependent expression of nuclear-localized β-Galactosidase (nlacZ) and GFP-GluA1(TG) from the bidirectional promoter (P_tet-*bi*_) controlled tTA responder operon. Strong transgenic GFP-GluA1(TG) expression in hippocampal layers (CA1, CA3, DG) is visualized by GFP-fluorescence in brain sections of *Tg8.1* mice. Scale bar, 500 μm. (**B**, right) nEPSP slopes before and after tetanization (arrow) at hippocampal CA1 synapses in controls (wild type, black-filled circles) and *Gria1*^−/−^*/Tg8.1* (green-filled circles) mice. Right, LTP was restored in *Gria1*^−/−^*/Tg*^*GFP-GluA*1(*TG*)^ mice. Although significantly reduced (*p* < 0.05) when compared to LTP in wild-type mice LTP in *Gria1*^−/−^*/Tg*^*GFP-GluA*1(*TG*)^ mice was well-developed. Numbers of experiments (*n*) are indicated.

Activity-induced changes in synaptic responses at CA3-to-CA1 synapses were assessed in acute brain slices of adult mice using cellular- and field-recordings (Figures 2, 3). In slices of control mice, low frequency stimulation (0.67 Hz for 3 min) at presynaptic sites in *str. radiatum* paired with depolarization (at 0 mV) in voltage-clamp, whole cell recordings of hippocampal CA1 neurons elicited a robust and long-lasting potentiation of excitatory postsynaptic currents (EPSCs) compared to the un-paired control pathway in *str. oriens* (pairing-induced LTP after 20 min vs. control pathway, wild type = 2.28 ± 0.20 vs. 1.24 ± 0.13, *p* < 0.01). Consistent with previous observations (Jensen et al., 2003), CA1 neurons in slices of adult *Gria1*^−/−^ mice did not express a significant pairing-induced LTP (1.18 ± 0.06 vs. 1.00 ± 0.14, *p* > 0.3; Figure 2). However, after additional genetic removal of GluA2 the remaining AMPARs in pyramidal CA1 neurons lacking both, GluA1 and GluA2, were sufficient to produce pairing-induced LTP in hippocampal slices of *Gria1*^−/−^*/2*^Δ*Fb*^ mice (1.67 ± 0.1 vs. 1.13 ± 0.1, *p* < 0.01). Moreover, expression of GluA2(Q) in *Gria1*^−/−^*/2*^*QFb*^ mice enabled potentiation of CA1 EPSCs (2.07 ± 0.14 vs. 1.05 ± 0.09, *p* < 0.01) which was similar to pairing-induced LTP of control mice (Figure 2).

In hippocampal field recordings using the tetanization paradigm (100 Hz, 1 s) we could also monitor LTP in *Gria1*^−/−^*/2*^Δ*Fb*^ and *Gria1*^−/−^*/2*^*QFb*^ mice (Figure 3A). Field excitatory postsynaptic potentials (fEPSPs) in the tetanized pathway were significantly increased when compared to the non-tetanized control pathway (normalized fEPSP slopes 45 min after LTP induction), both in *Gria1*^−/−^*/2*^Δ*Fb*^ (1.19 ± 0.05 vs. 1.04 ± 0.03, *p* < 0.01) and in *Gria1*^−/−^*/2*^*QFb*^ (1.21 ± 0.04 vs. 1.00 ± 0.02, *p* < 0.01; Figure 3A).

Similarly, we obtained an LTP rescue in *Gria1*^−/−^ mice that express a transgenic, PDZ motif-truncated and GFP-tagged GluA1(TG) mutation (Freudenberg et al., 2013a) in excitatory neurons of the forebrain (*Gria1*^−/−^*/Tg*^*8.1*^). In these mice the αCaMKII promoter-driven transgene *Tg*^*aCaMKII-tTA*^ (Mayford et al., 1996) permits the cell-type specific GFP-GluA1(TG) expression (Figure 3B, left). Hippocampal LTP of *Gria1*^−/−^*/Tg*^*8.1*^ mice was well-developed (1.29 ± 0.04 vs. 1.01 ± 0.02; *p* = 0.01), but was still significantly reduced (*p* < 0.05) when compared to LTP of wild-type mice (1.47 ± 0.05 vs. 1.03 ± 0.01; *p* = 0.01; Figure 3B, right). Importantly, the field-LTP in *Gria1*^−/−^*/Tg*^*8.1*^ mice reached a potentiation level that was monitored in slices of *Gria1*^−/−^*/2*^Δ*Fb*^ and *Gria1*^−/−^*/2*^*QFb*^ mice and that was achieved by the transgenic, full-length GFP-GluA1 expression (Mack et al., 2001). But despite the higher transgenic GFP-GluA1(TG) expression levels compared to GFP-GluA1 (Freudenberg et al., 2013a) and comparable LTP, the SWM of in *Gria1*^−/−^ mice was only observed in GFP-GluA1-, but not in GFP-GluA1(TG)-expressing GluA1 knockout mice (Schmitt et al., 2005; Freudenberg et al., 2013a,b). Similarly, the forebrain-specific GluA2 knockout mice (*Gria2*^Δ*Fb*^) developed regular levels of LTP but showed strong SWM deficits (Shimshek et al., 2006).

### Spatial working memory in GluA1-deficient mice with genetically recovered LTP

The lack of SWM in *Gria1*^−/−^*/Tg*^*8.1*^ mice and *Gria2*^Δ*Fb*^, despite the presence of partial or full LTP (Shimshek et al., 2006; Freudenberg et al., 2013b) led us to study the SWM performance of *Gria1*^−/−^*/2*^Δ*Fb*^ and *Gria1*^−/−^*/2*^*QFb*^ mice. We tested *Gria1*^−/−^*/2*^Δ*Fb*^ and *Gria1*^−/−^*/2*^*QFb*^ mice together with control and *Gria1*^−/−^ mice as negative control in the rewarded alternation task on a T-maze. Control mice alternated efficiently and visited the previously blocked target arm in the test run (77.6 ± 3.1) while *Gria1*^−/−^ performed not different to chance level (53.4 ± 1.6) as reported (Reisel et al., 2002).

**Figure 4:**
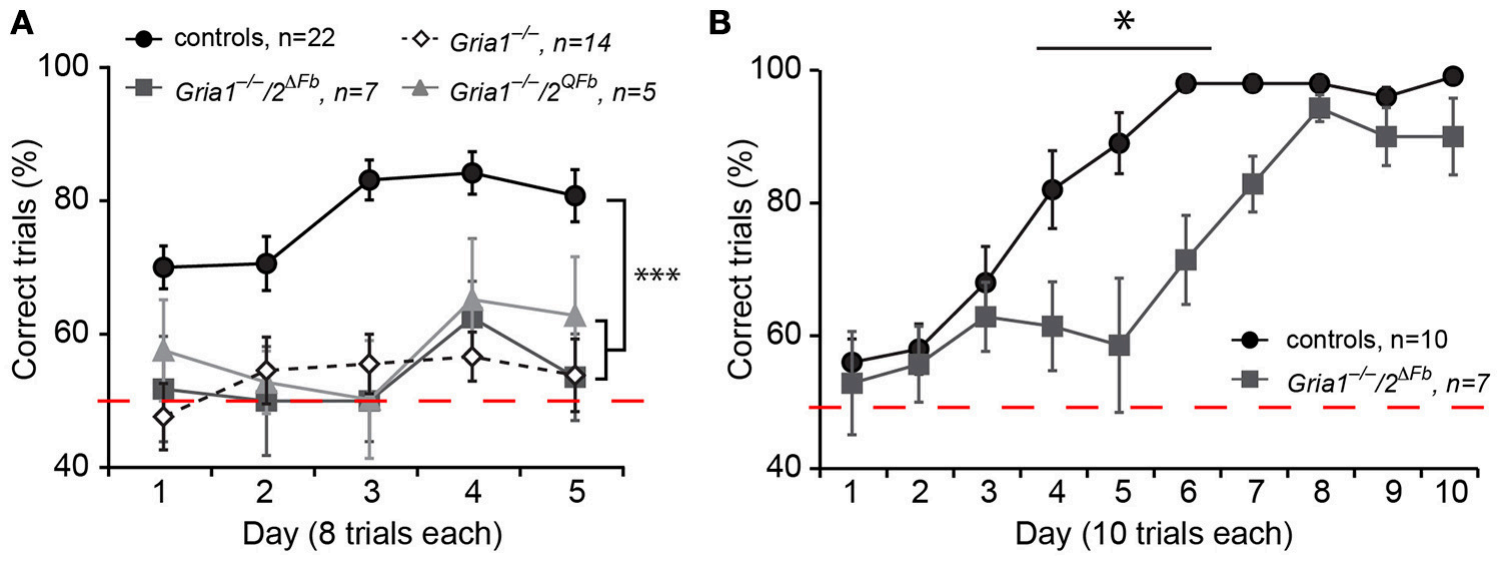
SWM impairment in *Gria1*^−/−^*/2*^Δ*Fb*^ and *Gria1*^−/−^*/2*^*QFb*^ mice. **(A)** *Gria1*^−/−^*/2*^Δ*Fb*^ and *Gria1*^−/−^*/2*^*QFb*^ mice exhibit impaired performances in a non-matching-to-place alternating T-maze. While control mice (black circles) alternate efficiently, both genotypes (*Gria1*^−/−^*/2*^Δ*Fb*^, dark-gray filled squares; *Gria1*^−/−^*/2*^*QFb*^, light-gray triangles) perform at chance level as observed in *Gria1*^−/−^ mice (white-tilted open squares). Performance is measured in percentage of correct trials. Numbers of tested mice (*n*) are indicated. Data in mean ± SEM; ^***^p < 0.005. **(B)** *Gria1*^−/−^*/2*^Δ*Fb*^ mice acquire SRM for a milk reward according the matching-to-place paradigm on an elevated Y-maze. SRM acquisition is delayed by 3 days in *Gria1*^−/−^*/2*^Δ*Fb*^ mice (dark-gray filled squares) when compared to controls (wild type, black filled circles). The performance is given as % correct trials. Numbers of tested mice (*n*) are indicated. Data in mean ± SEM; ^*^p < 0.05.

Regardless of the activated expression of endogenous Ca^2+^-permeable AMPARs and the restored LTP at CA3-to-CA1 synapses of *Gria1*^−/−^*/2*^Δ*Fb*^ and *Gria1*^−/−^*/2*^*QFb*^ mice, both lines displayed in the rewarded alternation task on the elevated T-maze a blunted SWM comparable to that of *Gria1*^−/−^ mice (*Gria1*^−/−^*/2*^Δ*Fb*^, 53.6 ± 2.3; *Gria1*^−/−^*/2*^*QFb*^, 57.5 ± 2.8; five blocks of eight trials, correct trials in %; mean ± SEM; Figure 4A). Repeated measures of two-way ANOVA revealed a main effect of genotype [*F*_(3/44)_ = 22.51, *p* < 0.0001] and block [*F*_(4, 176)_ = 2.61; *p* < 0.04], but any genotype-by-block interaction [*F*_(12, 176)_ = 0.73; *p* > 0.72]. *Post-hoc* Student-Newman-Keuls comparison identified significant differences (*p* < 0.05) for all three mouse models (*Gria1*^−/−^, *Gria1*^−/−^*/2*^Δ^^*Fb*^, Gria1^−/−^*/2*^*QFb*^) vs. control mice while mutants did not differ among each other. In addition, one sample *t*-test to the theoretical mean of 50% SWM performance (chance level) revealed significant values (*p* < 0.05) for control mice while all mice of the GluA1-deficient mouse lines did not perform differently from chance level.

*Gria1*^−/−^*/2*^Δ*Fb*^ mice were also tested to learn a fixed location of an arm in a Y-maze in 10 blocks of 10 trials each (Figure 4B). *Gria1*^−/−^*/2*^Δ*Fb*^ mutants were able to find the milk reward in the designated target arm efficiently and similar as control littermates (block 10: 90.0 ± 5.8 vs. 99.0 ± 1.0, *p* > 0.19), supporting the finding of *Gria1*^−/−^ mice that the SWM is not a prerequisite for the formation of SRM (Reisel et al., 2002; Sanderson et al., 2009). However, as also observed in *Gria2*^Δ*Fb*^ mice (Shimshek et al., 2006), SRM acquisition in *Gria1*^−/−^*/2*^Δ*Fb*^ mice was delayed and showed a lower success rate on day 4, 5, and 6 (*p* < 0.05, *p* < 0.001, *p* < 0.001, respectively; Bonferroni *post-hoc* test) indicating a specific role of GluA2 for certain behaviors.

## Discussion

In our study we used genetically modified *Gria1* and *Gria2* genes to modulate hippocampal AMPAR expression in GluA1-deficient mice. The cell-type specific modulation of AMPARs was achieved by inactivating a floxed *Gria2* gene, by activating a hypomorphic *Gria2*^*neo*^ gene and by expressing a transgenic GFP-tagged-GluA1(TG) in principal forebrain neurons of GluA1 knockout mice. In the three different mouse lines—*Gria1*^−/−^*/2*^Δ*Fb*^, *Gria1*^−/−^*/2*^*QFb*^, and *Gria1*^−/−^*/Tg*^*8.1*^—the remaining AMPAR levels and the ratios of Ca^2+^-permeable and Ca^2+^-impermeable AMPARs is very different in principal neurons of the hippocampus.

In hippocampal neurons of *Gria1*^−/−^*/2*^Δ*Fb*^ mice, the GluA3 level, was about 25% lower compared to GluA3 levels of wild-type mice, where GluR3 subunits already represent only 10% of the AMPAR subunits (Wenthold et al., 1996; Lu et al., 2009). In *Gria1*^−/−^*/2*^*QFb*^, which express both GluA2 and GluA2(Q), the fall in GluA3 expression was less pronounced than in *Gria1*^−/−^*/2*^Δ*Fb*^ mice even though the difference reached no statistical difference (*p* = 0.22). This might suggest that AMPARs containing only Glutamine (Q) in the pore-forming segment (Sprengel et al., 2001) are less stable and might be faster degraded than Ca^2+^-impermeable channel assemblies containing GluA2 with an Arginine (R) at homologous position. Similarly the two-fold reduction of GluA2 levels in *Gria1*^−/−^*/2*^*QFb*^ mice is less pronounced when GluA1 is present in *Gria2*^*QFb*^ (also called *Gria2*^Δ*ECS*^) mice, as demonstrated in an earlier study (Feldmeyer et al., 1999). On the other hand, we cannot exclude changes in *Gria2* and *Gria3* gene expression in response to GluA1 depletion.

The immunohistological analysis of coronal brain slices confirmed the absence and reduced GluA2 expression in *Gria1*^−/−^*/2*^Δ*Fb*^ and *Gria1*^−/−^*/2*^*QFb*^ mice, respectively. In addition, the somatic accumulation of GluA2 immunosignals in the *str. pyramidale* of *Gria1*^−/−^*/2*^*QFb*^ mice showed that a substantial fraction of GluA2 is trapped in the cell somata. Despite the loss of synaptic AMPARs in *Gria1*^−/−^*/2*^Δ*Fb*^ and *Gria1*^−/−^*/2*^*QFb*^ mice, the recorded I/V curves of CA1 pyramidal cells documented the contribution of the remaining AMPAR subunits in fast synaptic signal transmission. As expected from the expression analysis, the AMPAR currents in CA1 cells were strongly reduced when GluA1 and GluA2 were not expressed in *Gria1*^−/−^*/2*^Δ*Fb*^. The remaining GluA3-containing AMPAR in CA1 cells of *Gria1*^−/−^*/2*^Δ*Fb*^ mice could be identified by a high rectification index (RI)—the hallmark of Ca^2+^-permeable AMPARs (Burnashev et al., 1992). In CA1 pyramidal neurons of *Gria1*^−/−^*/2*^*QFb*^ mice the presence of GluA2(Q) in AMPAR assemblies could also be monitored by the formation of synaptic Ca^2+^-permeable AMPARs, as shown by the small but significant shift of the RI compared to the RI monitored in wild-type mice; the AMPAR-mediated current amplitude was similar to wild type.

The expression of endogenous encoded AMPARs in *Gria1*^−/−^*/2*^Δ*Fb*^ and *Gria1*^−/−^*/2*^*QFb*^ mice was sufficient for the induction and expression of pairing-induced and field-LTP in GluA1-deficient mice. However, the different amount of AMPARs affected the potentiation level. The GluA3-containing AMPARs of *Gria1*^−/−^*/2*^Δ*Fb*^ mice showed slightly lower LTP levels compared to the partial LTP rescue of *Gria1*^−/−^*/2*^*QFb*^ mice. A partial recovery of field-LTP in *Gria1*^−/−^ mice was also achieved by the transgenic GFP-GluA1(TG) subunit in *Gria1*^−/−^*/Tg*^*8.1*^ mice confirming that the GluA1-PDZ domain is dispensable for LTP (Kim et al., 2005). Thus, for the pairing-induced and field-LTP, there is no strict requirement for functional GluA1 subunits, but the pool of extracellular iGluRs affects the level of potentiation as described earlier (Granger et al., 2013).

Despite the partially restored hippocampal LTP in our three mouse lines, the SWM performance of all three lines remained at the chance level in the T-maze task. The lower amplitudes of LTP are unlikely to be the main reason for the failure to rescue the SWM impairment of GluA1 knockout mice. As we described earlier a partial LTP rescue with similar amplitudes obtained by the transgenic GFP-tagged-GluA1 expression was sufficient to improve the SWM performance in *Gria1*^−/−^ mice (Mack et al., 2001; Schmitt et al., 2005) whereas a fully developed LTP in forebrain-specific GluA2 knockout mice (*Gria2*^Δ*Fb*^) was associated with strong SWM impairment (Shimshek et al., 2006). Therefore, we conclude that the hippocampal LTP cannot be used to predict the behavioral performance of mice. Their SWM performance might be influenced by many factors modulating the excitatory and inhibitory systems, which might be more important than experimentally induced synaptic plasticity.

## Author contributions

DS, TB, LL, and RS designed, generated, and molecularly analyzed the mouse lines. VJ, BS, and GK performed and analyzed the electrophysiological experiments. VM and DS performed the behavioral experiments. RS, DS, and TB wrote the manuscript.

### Conflict of interest statement

DS and BS are currently employed by Novartis Pharma AG, Basel, Switzerland. However, this work was completed whilst DS and BS were employed at the Max Planck Institute for Medical Research. The other authors declare that the research was conducted in the absence of any commercial or financial relationships that could be construed as a potential conflict of interest.
