# Supplementary material for: Different Forms of AMPA Receptor Mediated LTP and Their Correlation to the Spatial Working Memory Formation
Source: Front Mol Neurosci. 2017 Jul 4;10:214. doi: 10.3389/fnmol.2017.00214 (PMC5495865; doi:10.3389/fnmol.2017.00214)
Supplement: Supplementary file 1 [file DataSheet1.DOCX]

Supplementary Material

Different forms of AMPA receptor mediated LTP and their correlation to the spatial working memory formation

**Derya R. Shimshek^+1^, Thorsten Bus^+1,2^, Bettina Schupp^1^, Vidar Jensen^3^, Verena Marx^1,4^, Liliana E. Layer^1,5^, Georg Köhr^1,6^ and Rolf Sprengel^1,2*^**

*** Correspondence:** Rolf Sprengel: [Rolf.Sprengel@mpimf-heidelberg.mpg.de](mailto:Rolf.Sprengel@mpimf-heidelberg.mpg.de)

# Supplementary Figures


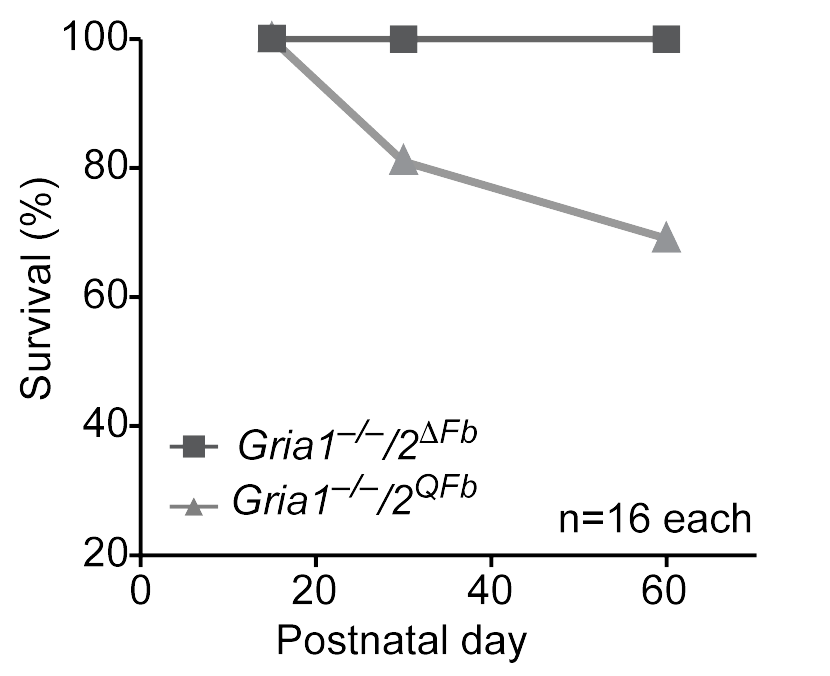


**Supplementary Figure 1. Increased survival by additional *Gria1^–/–^* gene manipulation in *Gria2^QFb^* mice.** Survival curves for *Gria1^–/–^/2^∆Fb^* (squares, dark grey) and *Gria1^–/–^/2^QFb^* mice (filled triangles, grey).


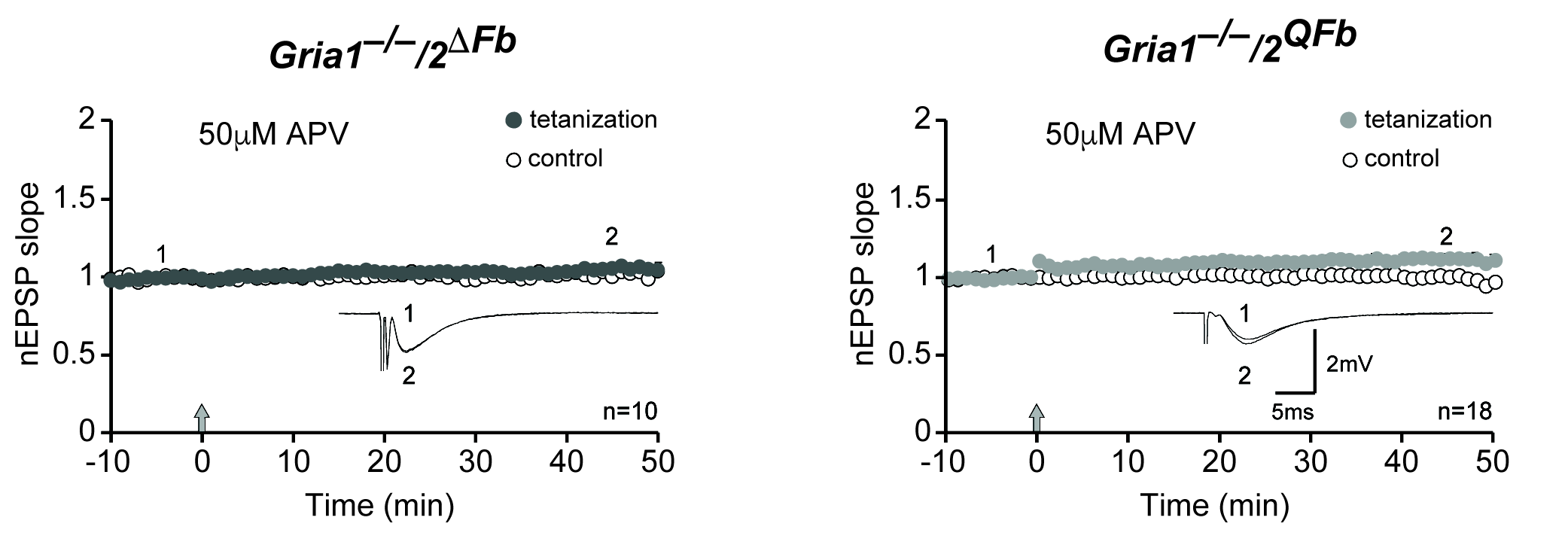


**Supplementary Figure 2.** **NMDAR-dependence of hippocampal field-LTP in *Gria1^–/–^/2^∆Fb^* and *Gria1^–/–^/2^QFb^* mice**. Normalized slopes of excitatory postsynaptic potentials (nEPSP slopes) before (1) and 45 minutes after (2) tetanization (arrows) to stimulation pathways (filled circles) in comparison to nEPSP slopes of un-tetanized control pathway (open circles). To block NMDAR activity, experiments were performed in presence of D-AP5 (50 µM). Insets: mean of six consecutive synaptic responses from single experiments. Scale bars, 5 ms, 2 mV.
